# Integrating Environmental Sustainability Considerations into Food and Nutrition Policies: Insights from Australia’s National Food Plan

**DOI:** 10.3389/fnut.2015.00029

**Published:** 2015-09-17

**Authors:** Ella Megan Ridgway, Mark Andrew Lawrence, Julie Woods

**Affiliations:** ^1^School of Exercise and Nutrition Sciences, Deakin University, Burwood, VIC, Australia

**Keywords:** environmental sustainability, public consultation, national food and nutrition policy, policy-making, stakeholder interests

## Abstract

The environmental sustainability (ES) of food systems is a critical challenge for policy makers. This is a highly contested policy area with differing views among stakeholders. The aim of the study was to develop a better understanding of how ES considerations are addressed in Australian food and nutrition policies and the way that consultation processes affect final policy outcomes. A mixed-methods study design combined a detailed chronology of key policy developments (2009–2015), a content analysis of written submissions obtained during the NFP’s consultation period (2011–2013) and a frame analysis of the sustainability perspectives – efficiency, demand restraint, and system transformation – in the NFP’s Issues, Green, and White Papers. There were 555 written submissions responding to two consultation papers. Stakeholders represented all sectors of Australia’s food system including government, non-government organizations, the food supply chain, research and academic institutions, and members of the general public. Around 74% of submissions referred to ES considerations and ~65% supported their inclusion into the final policy. Efficiency frames were most dominant; emphasizing a production-oriented approach that regards the environment as a natural resource base for food production but overlooks consumption and equity concerns. Despite strong support for the inclusion of ES considerations in the NFP, the influence of Australia’s socio-political context, powerful, industry-dominated stakeholders, and a reliance on traditional production-oriented perspectives delivered a business-as-usual approach to food policy making. It has since been replaced by an agricultural strategy that provides only cursory attention to ES. Our findings indicate that Australia’s political environment is not sufficiently mature for ES considerations to be integrated into food and nutrition policies. We propose reforms to the current consultation process in Australia to better support this integration by promoting greater transparency and participation in the development of food and nutrition policy making.

## Introduction

The promotion of healthy and sustainable diets is now recognized as a priority for food and nutrition policy (1–3). The consumption of excess energy has contributed to an increased prevalence of overweight (1.9 billion people) and obesity (600 million people) worldwide, further increasing demands for food (4). Nutrition-related chronic diseases like heart disease and diabetes are becoming more prevalent and are now one of the leading causes of death in high-, middle-, and low-income countries (5). Estimates suggest that global food production will be required to double in order to support a global population of 9.7 billion by 2050 (5). This will place a substantial demand on the food system’s natural resource base and its ability to continuously provide sufficient food into the future. A bidirectional relationship mediated via the food system exists between environmental sustainability (ES) and public health nutrition (6). The structure and operation of the food system influences the use of finite natural resources and the production of environmental waste (7). Equally, environmental resources, such as water availability and soil nutrients, influence food system outputs including the yield, availability, and quality of food production (8). Particular ES criteria for food and nutrition policies to consider in redesigning food systems have been identified to help promote healthy and sustainable diets. These criteria include reducing excess food energy consumption; reducing intake of energy-dense, nutrient poor foods; reducing food waste; and promoting plant-based diets (9).

A dichotomy has developed in contemporary food and nutrition policy making where *food* policy’s role is to regulate the production, distribution, and consumption of food whereas *nutrition* policies promote population health through good nutrition (10, 11). The “productionist” paradigm has represented the dominant model for food systems in developed countries since the post-war period, including Australia (10). This approach relies on science and technology to create intensive, high-yield agriculture and food production with deregulation to promote access to local and international markets (10). Food is positioned as a commodity and nutrition as an unrelated determinant of health. Food production, nutritional adequacy, and public health are driven further apart by dealing with the issues under separate government departments with different strategic goals (10). Given that the nutritional health of a population is so important to its socio-cultural and socio-economic prosperity, public health nutrition seeks to promote and maintain nutrition-related health and wellbeing through a coordinated set of activities and programs (12). The New Nutrition Science provides a more holistic model for public health nutrition by integrating environmental and social dimensions with the biological, and thus the promotion of food security and food system sustainability (13). However, Carlisle and Hanlon describe a “schism” between the quality of evidence, range of problems, and capability of current thought and practice to create effective solutions to public health challenges in affluent societies (14). They ascribe this to the nature of contemporary health issues like food system sustainability, often described as “wicked problems.” These are contentious issues that are difficult to resolve through policy because of their “incomplete, contradictory, and changing requirements” (15).

When a policy takes a particular stance about a contested issue, it may be better understood through an analysis of the surrounding discourse. Discourses order the way that “policy actors perceive reality, define problems, and choose to pursue solutions” to further their particular bias (16, 17). The use of discourses in this way is referred to as framing. Framing theory has been developed as a theoretical approach to help explain the powerful influence of stakeholders in policy making (18). Policy actors negotiate their legitimacy and assert power in the policy-making process by making claims about which interests they represent and by interpreting problems and evidence through a particular lens or perspective that promotes their agendas (18, 19). Frames can play a major role in the exertion of political power by identifying which interests dominate the debate. Consensus frames arise when a term gains broad resonance and consent among various groups who may have contradictory policy positions (20). For example, the need for sustainability (in its broadest sense, which includes economic, societal, and the environmental dimensions) is well supported across most sectors of society (21). However, there are many different food philosophies; some view the food system through an economic or market-oriented lens, while others are more concerned with the population and their health (22). As a result, the ES of the food system is a value-laden policy issue. Stakeholders can appropriate the sustainability term and attach broader meaning to it, creating a “fractured consensus” (20). The lack of action on sustainable food consumption and production by policy makers has been attributed in part to the “fuzzy and ill-defined nature” of food system sustainability (23).

International developments in food and nutrition policy have made positive steps toward a greater regard for ES considerations. The United Nations’ “Sustainable Development Goals” promote an integrated approach to food policy as well as sustainable consumption and production to enable “the protection and management of natural resources” (24). At a national level, Brazil released innovative dietary guidelines emphasizing the central role of socially and environmentally sustainable food systems, as have Qatar and Sweden (25–27). The advisory committee guiding the development of dietary guidelines in the United States also recently released a preliminary report emphasizing the need for environmentally sustainable production and consumption patterns, generating much debate and media attention (28). In the Australian context, political attention and support for the inclusion of ES matters in national food and nutrition policies has waxed and waned. In 1992, the Australian Government published the “National Food and Nutrition Policy” (29). An integrated approach was recommended to address five key issues, including ecological sustainability. It posited that the food system should be “both economically viable – and indeed contribute to the economic growth and performance – and maintain the quality and integrity of the environment” (29) Investment in the policy’s implementation was short lived and long-term interventions to help protect the sustainability of Australia’s food system were not established (30). Although the policy has never technically been rescinded, it has since become redundant.

For a considerable time, there has been a movement toward the integration of ES considerations in food and nutrition policies. As with other contested or wicked policy problems, we must first understand how policies are made in order to strengthen them and make progress toward achieving more integrated outcomes. There is a need to challenge dominant ideologies that drive the food system and overcome the disparate manner in which current policies have developed (31). This may be achieved through policy research by drawing on the inductive learning techniques that are typical of the social sciences. The development of Australia’s “*National Food Plan – Our Food Future*” (NFP) provided a rare opportunity to explore the development of a National Food and Nutrition Policy. The aim of our research was to develop a better understanding of how ES considerations are addressed in Australia’s food and nutrition policy making by focusing on how consultation processes affect the final policy outcome. Our objectives were to investigate the development of the NFP from an historical perspective and consider the political context throughout that time; identify the number and type of stakeholders engaging in the NFP’s development; determine the level of stakeholder support for the integration of environmental considerations into the NFP; and to identify how ES considerations were framed in key policy documents and any change over time as well as to pilot an innovative method to analyze the development of a food policy.

## Materials and Methods

### Study design

To achieve our aim, we needed to be able to reveal and expose the particular barriers and drivers to integrating ES considerations into the NFP. We developed an innovative method for analyzing the policy-making process that emerged from the particular nature and context of the NFP’s development. A standard practice in developing study designs and methods for policy research is to combine and adapt qualitative and quantitative techniques (32). We implemented a mixed-methods approach that combined a chronology of key policy developments from 2009 to 2015 and a longitudinal content analysis of written submissions from NFP stakeholders obtained during the consultation period (2011–2013). The chronology described the context of the NFP’s development, key events, and documents, when and where stakeholders engaged with the debate and the dynamics, sequence and interrelation between these and the NFP’s development. Longitudinal content analyses allow the researcher to scrutinize textual data for overt and covert messages, with an emphasis on the sender and the audience (33–35). Our content analysis examined the semantic representations present in all publicly available written submissions responding to the NFP’s Issues Paper and Green Paper. This analysis identified the types of stakeholders contributing to the NFP’s consultation processes according to their interest in the food system, whether ES was acknowledged, if the author supported its inclusion in the final policy and any change over time between iterations of the NFP. The NFP’s Issues, Green, and White Papers underwent a frame analysis using Garnett’s three perspectives for food system sustainability – efficiency, demand restraint, and system transformation (36). A validation step and an auditability trail were built into the study design so that the findings from each type of data, method, and investigator could be crosschecked with another to ensure dependability and transparency (37).

### Data collection

Data collection revolved around the Australian Government’s publication of the “*Issues paper to inform the development of a national food plan*” (Issues Paper) in 2011, the “*National Food Plan Green Paper*” (Green Paper) in 2012 and the final White Paper in 2013. We applied a snowballing technique to Google Advanced, searching for gray literature relating to the NFP using basic operators and the ability to search entire domain names, specific types of websites, and documents (38). This is an appropriate and rigorous technique for this type of data source. The gray literature search was saturated around documents and events that brought the policy onto the political agenda and which included stakeholders who had provided written submissions or who had participated in other NFP consultation methods. We began by searching the National Department of Health and Ageing and Department of Agriculture, Fisheries and Forestry websites for the search terms “food polic*” *AND*/*OR* “nutrition* polic*,” “national food plan,” “environ* sustainab*”. Individuals, groups, and organizations emerged as documents were collected and the process was repeated for each new stakeholder website. Data was also collected in relation to the other public consultation processes that occurred during the development of the NFP. This additional material included secondary data from roundtable consultations, public meetings, and social media discussions that were undertaken. This data did not undergo further analysis due to feasibility and study design constraints.

The Australian Government’s Issues Paper, Green Paper, and NFP were collected along with all 555 publicly available written submissions generated during the policy’s consultation (39–41). The submissions and various policy papers were originally located on a website hosted by the Australian Government’s Department of Agriculture, Fisheries and Forestry. The website was archived on July 19, 2013 and is now available through the *National Library of Australia’s Trove web archive* (42). Submissions were not published on the website if they were provided in confidence, where the author did not authorize publication, where the submission contained defamatory material, offensive language or infringed on copyright principles (43). These were not sampled and it was unknown if their omission would affect the outcomes of the study. Finally, specific text features were collected from each of the written submissions to the NFP Issues and Green Papers. Information identifying the stakeholder authoring or co-authoring each submission was collected for analysis by categorization.

### Data analysis

Data analysis was conducted in four separate but complementary phases. First, the data collected during the gray literature was collated and presented as a timeline depicting the period between 2009 and 2015. This period was selected because it includes the point at which discussions about a proposed NFP first entered Australia’s political agenda until present day. Individual written submissions were excluded from the timeline but were represented by the corresponding development paper, either the Issues or the Green Paper. Each event or document depicted on the timeline was tabulated. Details regarding dates, relevant stakeholders, and key content were recorded and stored in an auditability trail. Documents and events were validated by at least one other researcher to ensure that all key developments were included and to confirm relevance and significance. As this was an inductive process, several documents and events that appear on the timeline between 2014 and 2015 were collected and analyzed in real time, allowing for a more fluid and current analysis of the food and nutrition policy making context in Australia.

Categorizing stakeholders was a critical activity of the second phase. Categorization is “the process of dividing the world into groups of entities whose members are in some way similar to each other,” which establishes order in complex situations, such as Australia’s food and nutrition policy-making environment (44). Categorizing the authors of each submission identified the types of interests with a stake in Australia’s food system; frequency of each interest group providing written submissions; and changes to the composition of these stakeholders overtime. In this second phase, written submissions were identified and categorized according to their author’s interest in the food system. Categorization was an iterative process that evolved as submissions were grouped. We began by consulting Food Standards Australia New Zealand (FSANZ), an independent statutory body involved in Australian food and nutrition regulation. FSANZ consultation processes organize stakeholders into the categories of “consumers,” “public health professionals,” “industry,” and “government representatives” (45). In response to the data, we included an additional category for “individuals.” Sub-categories were developed to further refine the categorization. Letterhead, signatories, and self-identifying comments of submissions were scrutinized to identify the author. The final categorization of each stakeholder was recorded, tabulated, and stored in an auditability trail to ensure that results could be replicated. The frequency of submissions in each category and sub-category that mentioned ES considerations and the number of submitters supporting their inclusion in the NFP was then counted. This process was conducted by the primary researcher (Ella Megan Ridgway) and validated by other members of the research team (Mark Andrew Lawrence and Julie Woods). Where there was disagreement among the researchers about the grouping of each author, the researchers conversed on the matter until consensus was reached, a standard protocol in qualitative research.

The third analysis phase involved a longitudinal content analysis of all publicly available submissions. First, we determined if the stakeholder did or did not include ES considerations in their submission. Converting all submissions into PDF format and using the “Find” function in Adobe Reader to search for “ES” and “climate change,” “land use,” “water use,” “biodiversity,” and “food waste” achieved this. These key words were purposively selected by the team of researchers in response to the main ES-related themes under which the Australian Government directed their consultation questions. Some submissions were provided to the Australian Government in formats that did not allow this approach to be used. For example, hand written letters were scanned by the Government before uploading onto the hosting website. Textual analysis had to be conducted manually by reading each submission and highlighting the relevant search terms. Results were quantified and tabulated, then presented as a percentage of stakeholders who included ES considerations by category and sub-category to facilitate comparison between groups and change overtime. Key words appearing in contents lists, forewords, reference lists, glossaries, or appendices were not counted, only those present in the text corpus. We used open coding to assess whether the stakeholder supported the inclusion of ES in the NFP. By using the “Find” function to search for key words in the first step, we were able to isolate the sections of each submission that made reference to ES considerations. Each statement was coded according to whether it made positive or negative statements about environmental considerations and whether the submitter referred to them as a “goal,” “aim,” “target,” or requiring “intervention” through the NFP. An overall determination was made as to whether or not the stakeholder explicitly supported its inclusion in the policy. A primary researcher completed this phase (Ella Megan Ridgway). Julie Woods and Mark Andrew Lawrence selected a random sample of submissions to validate the findings by repeating the content analysis procedures. In the final content analysis step, each process was repeated for the various iterations of the NFP and a summary of the content in each policy paper was provided.

Finally, the sustainability frames applied by the Australian Government in each of the three policy papers were analyzed. Each of the food system sustainability perspectives is defined according to GHG emissions, biodiversity, food security, and nutrition. The values and ideologies behind the efficiency perspective include informed choice and smart consumption. Garnett describes this perspective as “freedom to consume” (36). A greed narrative underpins the demand restraint perspective, which limits growth and ultimately refers to “freedom from consumption” (36). Under the system transformation perspective, capacity building and fairer terms of trade are supported. The perspective promotes “freedom to self-determine” (36). These viewpoints are not mutually exclusive and may be used at different times and to varying degrees. We developed a frame matrix based on these four components and used open coding to analyze each policy document.

## Results

### Chronology

**Figure 1:**
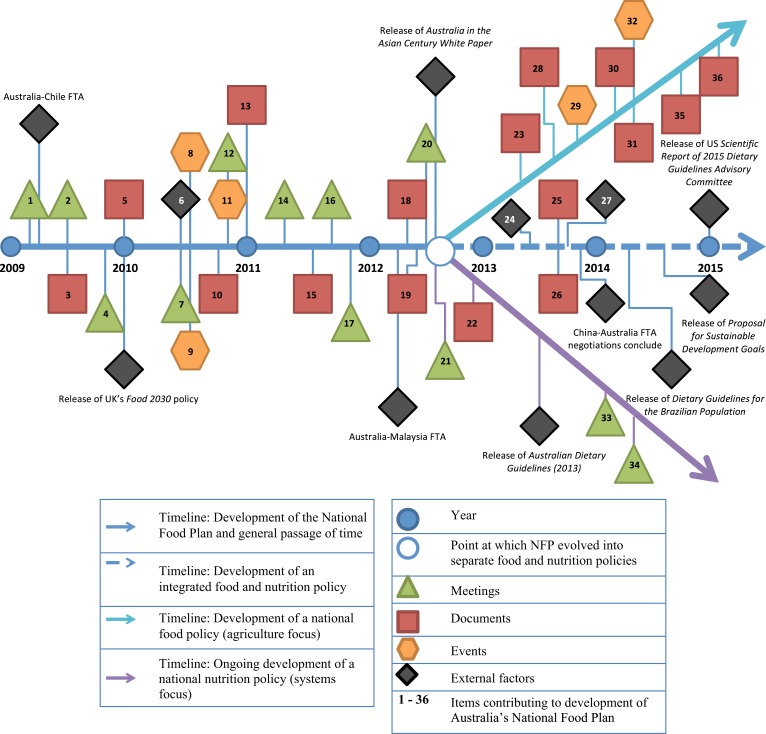
**Timeline of key activities during the development of the NFP (2009–2015)**. Systematic searching of gray literature sources produced key meetings, documents, events, and external factors contributing to the policy’s formulation, representing formal inputs by stakeholders into the policy-making process. This figure depicts the chronological development of the NFP between 2009 and 2015 with particular emphasis given to its evolution from an integrated policy into separate food (light blue arrow) and nutrition (purple arrow) policies.

Figure 1 sets out a timeline of the NFP’s developmental trajectory from 2009 to 2015, identifying the key stakeholders, documents, meetings, events, and external influences.

In 2009, the idea of a national food policy reappeared on Australia’s political agenda driven by the coordinated efforts of public health and food industry stakeholders. The Public Health Association of Australia (PHAA), Dietitians Association of Australia (DAA), Dairy Australia, Meat & Livestock Australia (MLA), the National Farmers Federation (NFF), and the Australian Food and Grocery Council (AFGC) were particularly active. Several meetings were held between public health and food industry groups, resulting in the publication of key documents to better understand “the impacts of food production on ES from a public health perspective” and establish “guiding principles for the implementation of a new whole of government National Food and Nutrition policy in Australia” [Items 1–5] (46, 47).

Stakeholders successfully advocated for the inclusion of a national food plan on the 2010 Federal Election agenda [Item 6]. The NFP’s development was announced after the election [Item 8]. The Federal Opposition also developed a “food security policy” [Item 9]. In October 2010, a landmark document, “Australia and food security in a changing world” [Item 10] substantiated the environmental and food security challenges for Australia (48). A National Food Policy Working group was established, comprising of 12 food industry leaders, a consumer advocate, and a nutrition representative [Item 11] (49). The Global Foundation, under the leadership of CEOs and executives from Woolworths, SunRice Australia, and Visy, also hosted a roundtable discussion with the Agriculture, Fisheries and Forestry Minister and a summit on food security [Item 7; Item12] (50).

In January 2011, the independent review of Australia’s food regulation and labeling environment, “Labeling Logic” [Item 13], called for a national nutrition policy for the provision of an “overarching framework … within the context of the government’s preventative health agendas” (51). The National Food Policy Working group convened a meeting in April, prior to the release of the Issues Paper in June 2011 [Items 14–15]. The Department of Agriculture, Fisheries and Forestry coordinated the Issues Paper in collaboration with several other government portfolios (39). Additional roundtable discussions were held between the Department of Agriculture, Fisheries and Forestry and The Global Foundation [Item 16] as well as a meeting of the National Food and Nutrition Leaders’ Science Forum, a collaborative body headed by the CSIRO [Item 17]. The Forum established a dialog between selected food and nutrition stakeholders with an emphasis on building a common vision for research and development across the food system (52).

The Green Paper was published in July 2012 [Item 18]. It outlined the Government’s approach to food policy and potential changes to policy, programs, and governance arrangements (40). At around the same time, the Australian Food Sovereignty Alliance (AFSA) published the “People’s Food Plan Working Paper” [Item 19] to challenge the dominant, production-based discourse and deliver a national food plan based on the principles of food sovereignty (53). Another meeting of the National Food and Nutrition Leaders’ Science Forum was convened in September 2012 [Item 20]. Two key events in October–November of 2012 signified the point at which the NFP ceased to be an integrated food and nutrition policy, marked by the point of divergence in Figure 1. Along with the Green Paper’s sidelining of nutrition, the Government confirmed the development of a national nutrition policy in response to recommendations from “Labeling Logic” in October 10 [Item 21] (54). A request for tender [Item 22] was released as part of the government’s scoping study to inform the policy (55). From this period onward, the NFP and the notion of a national nutrition policy were regarded as separate entities.

Australia’s NFP was released in May 2013 [Item 23]. Both the Australian Greens (political party) and AFSA continued to publish their own integrated food plans [Items 25–26] (56, 57). However, the newly elected Coalition Government abandoned the NFP and announced the development of an “Agricultural Competitiveness White Paper” in December 2013 [Item 29] (58). The CSIRO and National Food and Nutrition Leaders’ Science Forum’s work also culminated in a strategy for research, development, and technology transfer across the food system [Item 28] (52).

The Australian Bureau of Agriculture and Resource Economics and Sciences (ABARES) is the “research arm” of the Australian Government’s federal agriculture department. Their role is to provide independent research to both the government and private sector (59). In March 2014, the ABARES Outlook 2014 Conference [Item 32] included contributions from a number of business-interest organizations who had participated in the NFP’s consultation processes and who had a clear agenda in their business strategy for food security (60). The development of the now separate national nutrition policy included two further activities [Items 33–34]. The Government hosted an invitation-only meeting with 32 stakeholders from academia and research, public health, non-government organizations, food industry, and government. The Department of Health also established a National Nutrition Committee comprised of representatives from the State and Territory health departments and the Department of Agriculture, Fisheries and Forestry representatives to seek their input on the policy. The Committee met in April 2014. The deliberations at, and decisions from, these two meetings were not made publicly available. There has been no further activity to progress the development of a national nutrition policy (61). It would appear that the policy is no longer a priority for the Australian Government. An Issues Paper to inform the “Agricultural Competitiveness White Paper” was released for public consultation in February 2014 [Item 31] (58). This was followed by the publication of a Green Paper in October 2014 [Item 35], which did not discuss any ES considerations for the Australian food system. The Government stated that not all submissions would be made publicly available – some would not be published until after the consultation period ended (62). The final White Paper was published in July 2015 [Item 36]. Climate change and its consequences were framed as “particular challenges for sectors, such as agriculture, where profitability and productivity are closely linked to natural resources” (63).

### Consultation and stakeholder categorization

The written submissions responding to the NFP’s Issues and Green Paper represent the formal and comprehensive documentation of inputs into the policy-making process. This process generated 680 written submissions (*n* = 279 Issues Paper, 401 Green Paper submissions). All publicly available submissions were collected and analyzed in this study (*n* = 192 Issues Paper, 363 Green Paper submissions) and categorized by stakeholder type. Table 1 details the analysis of the absolute numbers and proportions of submissions for each stakeholder category and sub-category. Per category, non-government organizations submitted the most responses to the Issues Paper (*n* = 64 submissions, 33.3%), Green Paper (*n* = 129 submissions, 35.5%), and overall (*n* = 193 combined submissions, 34.8%). Government stakeholders represented the lowest number of Issues Paper submissions (*n* = 12 submissions, 6.8%). The lowest responses to the Green Paper were contributed by Research and Academic Agencies at 7.4 and 7.8%, respectively. Per sub-category, stakeholders from the Production sub-category in the food supply chain category contributed the greatest number of responses to the Issues Paper (*n* = 40 responses, 20.8%). The Green Paper results indicated that the highest frequency of submissions occurred in the lay person sub-category (*n* = 65 submission, 17.9%).

**Table 1:** **Number and frequency of written submissions per category and sub-category**.

Category	Issues Paper	% Total subs.	Green Paper	% Total subs.	Sub-category	Issues Paper	% Total subs.	Green Paper	% Total subs.
Government	13	6.8	40	11	International Federal State Local	0 2 5 6	0 1 2.6 3.1	1 2 7 30	0.3 0.6 1.9 8.3
Non-government organizations	64	33.3	129	35.5	Business interest Health Community and consumer Environment	11 8 26 20	5.7 4.2 13.5 10.4	23 17 45 44	6.3 4.7 12.4 12.1
Food supply chain actors	54	28.1	79	21.8	Production Processing Distribution Retail and marketing	40 10 3 0	20.8 5.2 1.6 0	58 19 1 1	16 5.2 0.3 0.3
Research and academic agencies	16	8.3	27	7.4	Independent research bodies Tertiary institutions Professional associations	3 7 6	1.6 3.7 3.1	8 10 9	2.2 2.8 2.5
Individuals	45	23.4	88	24.2	Professional Lay person	11 34	5.7 17.7	23 65	6.3 17.9
Total	192	100	363	100	Total	192	100	363	100

Overall, the number of submissions from stakeholders contributing to the NFP’s development increased over time with 34.6% of the total submissions responding to the Issues Paper and 65.4% responding to the Green Paper. This change over time was mirrored by a general increase in submissions per category when comparing the Issues Paper and Green Paper. All sub-category submissions increased between the Issues and Green Papers except for the Federal Government stakeholders. Only submissions from the distribution sub-category decreased between the Issues and Green Paper. Sixty-three stakeholders submitted to both papers making the number of submissions greater than the number of contributing stakeholders. Most stakeholders who submitted twice were from the non-government organizations (*n* = 22) and food supply chain actors categories (*n* = 20), with production stakeholders representing the highest number of multiple submissions by sub-category (*n* = 15).

The absolute number and proportion of stakeholders who acknowledged ES in their written submissions and went on to support its inclusion in the NFP are provided in Table 2. More than 74% of stakeholders submitting to the NFP Issues Paper and Green Paper acknowledged ES. In terms of whether stakeholders felt that this issue should be addressed in the NFP, around 65% of submissions would have supported a decision by the Australian government to include ES in the final NFP. Increases in the number and proportion of submissions acknowledging and supporting ES considerations increased overtime as a result of the overall increase in submissions between papers. Members of the general public, categorized under the lay person sub-category, were most likely to include ES in their Issues and Green Paper submissions (13.54 and 13.77%, respectively). All Issues Paper submissions from the lay person sub-category went on to support its inclusion in the policy (*n* = 26).

**Table 2:** **Number and frequency of written submissions acknowledging environmental sustainability considerations and supporting inclusion in NFP per category and sub-category**.

Stakeholder		Issues Paper				Green Paper			
Category	Sub-category	ES in sub.	% Total subs.	Want ES in NFP	% Total subs.	ES in sub.	% Total subs.	Want ES in NFP	% Total subs.
Government	International Federal State Local	0 1 6 5	0 0.5 3.13 2.6	0 1 6 3	0 0.5 3.13 1.56	1 2 7 25	0.3 0.6 1.93 6.89	1 2 6 22	0.3 0.6 1.65 6.06
Non-government organizations	Business interest Health Community and consumer NGOs Environment NGOs	8 3 19 20	4.17 1.56 10.42 10.42	4 3 15 18	2.08 1.56 7.81 9.38	16 9 31 41	4.41 2.48 8.54 11.3	14 8 29 40	3.9 2.2 8.0 11.0
Food supply chain actors	Production Processing Distribution Retail and marketing	20 8 3 0	10.42 4.17 1.56 0	19 7 2 0	9.9 3.65 1.04 0	41 9 0 0	11.3 2.48 0 0	32 8 0 0	8.82 2.2 0 0
Research and academic agencies	Research Academic Professional associations	3 3 11	1.56 1.56 5.73	3 3 10	1.56 1.56 5.21	7 8 7	1.93 2.2 1.93	6 7 5	1.65 1.93 1.38
Individuals	Professionals Lay person	6 26	3.13 13.54	5 26	2.6 13.54	17 50	4.68 13.77	17 36	4.68 9.92
Total		142	74.47	125	65.08	271	74.74	233	64.29

### Framing environmental sustainability in the NFP

The content of the NFP and the way that ES considerations were addressed changed overtime in conjunction with the consultation generated by the Issues and Green Papers. Table 3 demonstrates the Australian Government’s frequency of reference to each of the keywords identified by this study. Climate change appeared most frequently in the policy papers (*n* = 158), followed by food waste (*n* = 77), land use (*n* = 76), biodiversity (*n* = 37), and finally water use (*n* = 25).

**Table 3:** **Content analysis of key words in NFP policy papers**.

Policy document	Word count	Climate change	Land use	Water use	Biodiversity	Food waste	% Frequency (all key words per paper)
Issues Paper	45,764	44	12	5	9	7	0.2
Green Paper	88,562	82	40	13	19	41	0.2
White Paper	38,324	32	24	7	9	29	0.3
Total	172,650	158	76	25	37	77	0.2

All perspectives for food system sustainability were applied to some extent during the NFP’s development but as Table 4 demonstrates, the efficiency perspective was more dominant than demand restraint or system transformation – especially in the final White Paper. The main focus of attention for each of the policy papers was consistent with one or both of the efficiency and demand restraint perspectives of food system sustainability, and thus the productionist model for food production and consumption. The scope of the NFP Issues Paper included population growth, climate change and finite resources, the obesity epidemic, and burden of chronic illness as a result of poor nutrition and economic growth rates (39). The Green Paper did not mention ES considerations until Chapter 5, which referred to domestic food security (40). The White Paper set out an overall vision and four overarching themes under which goals were set for 2025. These included growing exports; a thriving industry; improving food security during natural disasters and for disadvantaged communities; developing a National Nutrition Policy; and sustainable food through the management of natural resources that affect food production capacity and consumption (41).

**Table 4:** **Frame analysis of food system sustainability perspectives in the NFP**.

	Efficiency	Demand restraint	System transformation
Focus of attention	Issues Paper, Green Paper, White Paper	Issues Paper, White Paper	
Climate change and GHGs	Issues Paper, Green Paper, White Paper		
Biodiversity	Issues Paper, Green Paper, White Paper		
Food security	Issues Paper, Green Paper, White Paper		Green Paper, White Paper
Nutrition	Issues Paper, Green Paper	Issues Paper, Green Paper	Issues Paper

Only the efficiency perspective was used by the NFP’s policy makers to frame the discourse around climate change and GHG approach and biodiversity. Here, greater food production and increased productivity with less environmental impact to “spare land for wilderness” is promoted (36). In the Issues Papers, competition for land use, climate change, and natural disasters were framed as “challenges” that slowed productivity whilst the influence of consumption patterns on the environment was omitted. The Issues Paper also regarded the capacity of the natural resource base and climate variability to “pose a challenge to ongoing agricultural productivity growth” (39). Climate change, degradation of the natural resource base, and natural disasters were positioned as challenges to industry competitiveness and productivity rather than population health in the Green Paper. A strong natural resource base was deemed essential for the profitability and productivity of primary industries and it was stated that the changing climate necessitated greater food production with fewer resources (40).

Initially, food security was defined under the efficiency perspective. In the Issues Paper, the Australian Government saw no foreseeable risk to food security (39). In the Green and White Papers, a combination of efficiency (increasing the global food supply) and systems transformation perspectives (equity of access) were applied. This may be due to the widespread recognition of the Food and Agriculture Organization’s food security definition, which is central to Garnett’s system transformation perspective through its recognition of the multidimensional nature of food security including access, affordability, utilization, and stability (36). Compared to the Issues Paper, the later papers acknowledged the importance of addressing food insecurity among disadvantaged populations.

Only the Issues Paper (which combined a combination of all three approaches) and Green Paper (combination of efficiency and demand restraint) included ES considerations relating to nutrition. Diet-related illness is a significant public health problem in Australia and this became a main focus of the policy papers especially with respect to meat and dairy consumption (demand restraint). Potential solutions to address chronic disease, food insecurity, and the ES of Australia’s food system revolved around crop fortification and product reformulation (efficiency). The Issues Paper made some mention of the benefits of local food systems especially with respect to food waste and the effects of food transport (system transformation), but this was omitted in later policy papers (39). The Green Paper described food waste as a contributor to chronic hunger whilst diet and nutrition were considered in the context of a safe food supply. However, it was noted that such issues would be addressed by a national nutrition policy and not by the final NFP (40).

## Discussion

Policy making is a dynamic process. Problem perception, shifts in elite, and public opinion about the salience of a particular problem, the iterative process of policy formulation and difficulties in policy reformulation have been identified as characteristics of the policy-making process (64). Sabatier’s model for policy making demonstrates that policy-making processes are influenced equally by macro factors like changing political, economic, and social conditions and the strategic interactions between the people within a policy-making community. This is referred to as a policy subsystem. As actors from a variety of institutions who are interested in the policy area compete for power and seek more knowledgeable ways to achieve their policy objectives, we see a shift in the way that the policy develops over time (64). Whilst this study sets out the development of Australia’s NFP in a linear way, its trajectory transformed as stakeholders entered and left the debate or asserted their power over the NFP and its ES objectives.

### The influence of external forces

Policy development exists within a broader socio-political context. Historically, Australia has developed a national identity tied to its food producing capabilities. Food exports are some of Australia’s most profitable because of the food system’s capacity to produce far more than is required domestically. The total food chain is worth ~$230 billion/year (65). The aggressive pursuit of deregulation and free trade has established a political environment that is dominated by neoliberal values and policies (36). Some scholars have attributed environmental degradation to neoliberalism, globalization, and social transformation (66). When this is the dominant political ideology within which a policy develops, there is a tendency for particular arguments and voices that support neoliberalism to overpower others. Analyzing texts and the interactions within and between them improves understandings of the processes through which government policies, especially those that facilitate neoliberalism, are created (67).

The events and documents leading to the final NFP did not occur in isolation or by chance. Lobbying, advocacy, policy development activities, and the broader socio-political context in Australia influenced the process. Meetings, events, public consultations, and publication of key documents intertwined to create the unique environment in which the NFP developed. Placing the idea of a national food plan on the political agenda was a result of the combined efforts of public health and food industry representatives, notably the PHAA and DAA. The loose collaboration acknowledged the importance of integrating food and nutrition objectives to promote both health and ES considerations. However, there was a failure to build on the common ground established between food industry and public health professionals so that ultimately groups representing growers and processors, such as the AFGC and National Farmers’ Federation, were satisfied with a more narrowly focused agricultural policy by the end of the NFP’s development.

Although there was disunity between groups of stakeholders and their broader interests in the food system by the end of the NFP’s development, early prioritization of ES considerations on the political agenda and overall support for their integration from NFP stakeholders highlights its importance as a policy issue in Australia. This was not reflected in the final NFP or by Australia’s broader policy environment. Policy action to address ES considerations in Australia appears to be moving further away from the approach originally proposed in 1992, despite the increasing urgency of the problem. The national *Food and Nutrition Policy* acknowledged the importance of government-wide action to promote “ecologically sustainable development” (29). The 3-year period during which it was implemented was characterized by health-centric interventions that failed to incorporate ES considerations in a way that influenced sustainable approaches to food security in the long term (30). Just as the influence of the health department’s ownership of the national *Food and Nutrition Policy* can be seen in its health-centric implementation, so too can the overriding sectoral influence of the Department of Agriculture, Fisheries and Forestry in the NFP’s focus on commercial and economic aspects of the food system. More recently, the proposed agricultural competitiveness policy has been developed as a food policy devoid of ES considerations. The term “sustainability” has been used many times in Australia’s broader political environment but with contrasting meanings. For example, the *Australia in the Asian Century White Paper*, which was published in 2012 alongside the NFP Green Paper, set out a national objective that “Australia’s agriculture and food production system will be globally competitive, with productive and sustainable agriculture and food businesses” (68). In this context, sustainability refers to an economically sustainable business model for the food system rather than the sustainability of the environment.

Australia has also signed onto a number of legally binding free trade agreements (FTAs). Under the auspice of the World Trade Organization, FTAs “liberalize” access to and investment in the markets of other countries. During the development of the NFP, Australia signed FTAs with Chile in 2009 and with Malaysia in 2012 (69). Once entered into effect, these FTAs have implications for agricultural export and foreign ownership of Australian agricultural land. In November 2014, negotiations concluded on the landmark China–Australia FTA removing tariffs on agricultural products and ensuring a greater advantage for the profitability of trade in this sector compared to Australia’s competitors. The agreement was signed in June 2015 (70).

The underlying circumstances and contexts of Australians’ food selection practices are an important consideration in the incorporation of ES considerations into food policies. Dixon and Isaacs identified a discrepancy between desired and actual behaviors in relation to the views of mainstream consumers toward healthy and sustainable diets (71). Even though consumers want to support Australian food suppliers, their food practices tend to be based preferentially on household budget and family nourishment. As a result, the economic conditions within Australian households must be addressed for ecological citizenship to be achieved. The authors also suggest that these practices place implications on the government’s “ecological authority,” which could be improved by supporting the normalization of sustainable practices like local food systems and community gardens (71).

### The influence of consultation

Public consultation is an important regulatory process that gives voice to stakeholders, allowing policy options to be tested and argued (72, 73). The intention of public consultation is to decentralize the power and influence of elite groups so that decision making is shared and mutually beneficial, improving transparency, efficiency, and effectiveness in policy making (74, 75). Transparency is a core value of democratic societies that holds governments accountable, exposing them to scrutiny and enhancing public trust (76). Effective consultation establishes a deeper understanding of the baseline conditions within which the policy will exist, identifies any unforeseen problems or issues of practicality and gages community reaction (72). Public experience, exposure to balanced views around the debate and involvement in policy development promote public support for complex policy issues. It is important for the government to create these opportunities (74).

A number of consultation methods were provided for stakeholders to engage with the NFP’s development. The number of written submissions indicated a high level of interest in the policy and an opportunity for the diverse range of interests to be heard. Engagement in the consultation processes tended to increase over time but the composition of stakeholder groups varied. The Australian Government successfully facilitated strong engagement from the broader public during consultation. Lay people were the most prevalent contributors of written submissions and to acknowledge and consider ES actionable under the NFP. Whilst the makeup of NFP stakeholders was diverse, food industry interests were particularly overrepresented. This was evident in the number of written submissions contributed by this sector and also in the composition of key groups designed to advise the Government and its direction for the plan, particularly the National Food Policy Working Group. This may have contributed to the way that the NFP progressed and the decision to minimize nutrition objectives in the final policy. Preference for particular interests was also evident between sub-groups under the same category. The production sub-category provided more submissions than processing, distribution, and retail and marketing, despite the fact that together food processing and retail sales contribute >80% of the food chain’s value (65). This may be a result of the emphasis that was placed on primary production in the consultation papers.

Categorization enabled the identification of otherwise “hidden” groups – stakeholders who previously appeared on the periphery of the debate or were not typically associated with food and nutrition policy making. These were predominantly from the business-interest NGO category, including The Global Foundation and Rabobank. These two stakeholders had strong agendas for furthering their sector’s interests around food security combined with strong ties to the government and financial resources and appeared to have greater access to the debate. A particularly strong example is the apparent access of The Global Foundation to government ministers, who called for a “food security policy” that considered a business-oriented approach rather than nutrition and the environment. Carey et al. who found that food and agriculture organizations and industry lobbies were given disproportionate influence over the way that the NFP was shaped support these findings (77). Critically, stakeholders from all sectors of the food system regarded ES as an important issue for the NFP. The way that submissions had been interpreted, weighted, and incorporated into the policy was not disclosed. Where stakeholder views are not included after consultation, efforts should be made to communicate and justify these decisions (75). These actions promote transparency in the decision-making process but were not taken by the Australian Government – a significant weakness of the consultation process as a whole.

### The influence of framing

The reliance on traditional and dominant frames, which stem from the principles of neoliberalism, meant that the policy evolved into a “business-as-usual” approach rather than spurring any significant transformation of the Australian food system. There is much debate around whether or not there is sufficient evidence to support the inclusion of sustainability measures in health policy more broadly. This creates a tension between the competing demands of policy making, which include political or financial imperatives, and absent or conflicting evidence from research (76). An insufficient or contradictory evidence-base is often used to justify the exclusion of sustainability principles from policies. Initially, the Australian Government planned to address the global and local forces impacting upon the country’s food system through a national food plan (39). The final NFP was more concerned with framing food production in a way that would promote economic prosperity. Very few direct actions to address the environmental condition of the food system were proposed and there was a noticeable shift between the three iterations of the NFP, with the early papers considering ES considerations more holistically. Despite more than half of the stakeholders supporting the inclusion of ES considerations in the NFP and the Government’s early intentions, the final NFP positioned sustainability in the context of maximizing food production for economic sustainability only. The final plan had an overt agricultural and food production focus combined with a significant proportion of food industry submissions and lack of publicly available information about the content of roundtable discussions and public meetings. This suggests that there was an elite group of stakeholders with inordinate influence on the policy-making process compared to other groups with equally valuable insights and solutions. Changes in emphasis on ES considerations and nutrition overtime, coupled with the way that the Issues and Green Papers guided the consultation process suggests a predetermined outcome for the policy, limiting the capacity for public health and non-food industry groups to influence the framing of ES considerations in Australia. The NFP applied all three of Garnett’s perspectives for food system sustainability, but addressed the efficiency perspective more completely. This became more dominant over time. A range of submitters challenged the government’s claim that there were no foreseeable risks to domestic food security but with nutrition dealt with by a potential nutrition policy, the final NFP did not set any directives or goals for population nutrition in the context of the food supply chain.

### Synthesizing the influences

The main reason that ES considerations were not integrated into the final NFP was the overriding influence of the prevailing political environment. The NFP’s consultation processes could be scrutinized because they were documented and made publicly available, with an open process for obtaining written submissions from interested parties. Our findings suggest that the majority of submissions called for policy action to address ES considerations in Australia’s food system and yet, this was not reflected in the final NFP. We were unable to identify how the Australian Government weighted stakeholder submissions and how the evidence provided by stakeholders was used to inform the policy. This uncertainty raises questions about whether this consultation process was a genuine attempt to canvas people’s views or whether it was merely an exercise in affirming a preconceived position. All stakeholder views may be valid and useful to decision making, but questions arise about the current policy consultation process in Australia and its robustness in capturing and responding to competing views. As Elgert suggests, we need to “establish a global, evidence-based discourse” that incorporates the multiplicity of perspectives on the matter and promote deliberative governance to limit the power of political elites (78).

This study has shown that despite the widespread support for the integration of ES considerations into the NFP expressed during the consultation process, the Australian Government chose to largely ignore this issue. There was very little evidence to suggest that the final NFP had taken on board key ES considerations like promoting plant-based diets and reducing consumption of excess energy and processed foods, as outlined in the introduction. Whilst it set out goals for reducing food insecurity and food waste, action was directed toward other policies that were set for future development rather than through the NFP itself. These included the National Nutrition Policy, Clean Energy Future Plan, and Carbon Farming Futures Policy all of which were made redundant after a change of Government in 2013. It was beyond the scope of this study to investigate whether participating stakeholders were able to directly influence the policy-making process and final policy outcome. Instead, what does emerge from the chronology is the influence of wider policy contexts on policy making in Australia. These policy contexts include the overarching neoliberal ideology that informs policy processes and the presence of powerful elites in the consultation process. The NFP’s development occurred at a time when Australia was positioning itself as the “food bowl” for Asia. Along with the *Australia in the Asian Century White Paper*, developing a globally competitive food industry through the promotion of free trade (especially with China) and deregulation was an overarching goal of the Australian Government’s policy making. In terms of the power exercised by stakeholder groups, our chronology identified an overrepresentation of stakeholders from food industry and business-interest NGOs on food policy working groups as well as increased access to government ministers through roundtable discussions and public meetings. The interests of these groups appeared to be regarded more highly than others, despite the fact that individuals represented the greatest number of submissions and highest levels of support for ES considerations. This supports the idea of a reliance on “technocratic models of decision making” in food and nutrition policy development, which allows powerful technical elites or “experts” to speak for the public (19). Our findings have provided important insights into Australia’s policy-making process by demonstrating the influence of these external forces over the policy process, raising a number of questions about the NFP and food and nutrition policy development more broadly. What is the purpose of consultation if the findings are not used to inform policy development? Is the interpretation of consultation responses affected or biased by the presence of powerful groups with more resources and a greater access to policy makers? What affect does this bias have on less powerful stakeholders who responded and their desire or capacity to continue to be involved in the policy-making process and other consultation opportunities?

To address these complexities, we propose a modified consensus model for consultation that incorporates two reforms to the current consultation process in Australia. The first is to enhance transparency by providing information regarding the assessment criteria and the way that submissions have been weighted in relation to the final policy outcome. For example, the government could prepare a summary report on the consultation processes that outlines how much significance is given to expert opinion as opposed to professional lobbying. Second, in acknowledging that access to the current consultation system is biased toward those who have the means to respond, participation by all interested individuals and groups needs to be further supported. This may entail the exploration of novel ways to increase the capacity, time and/or resources for marginalized groups, in particular, to contribute to the consultation process. For instance, the use of digital technology may improve access for those in geographically remote areas or could enhance literacy skills in those whose written communication skills are challenged. In combination, these two reforms will strengthen the robustness of the consultation process to help manage the political dynamics associated with efforts to incorporate sustainability into National Food and Nutrition Policy.

### Strengths and limitations

The opportunity to analyze food and nutrition policies is rare in Australia because of limited policy activity. The NFP provided a large body of data to interrogate. Where other studies have focused on the government, civil society, and the food industry, this study is unique in its analysis of all 555 publicly available submissions as well as its comprehensive coverage of all stages in the policy-making process with the chronology extending from 2009 to 2015 (77, 79). However, not all features of the NFP and associated gray literature could be analyzed by this study due to time and feasibility constraints. One shortcoming is the limited range of key words used in the content analysis. There are many other issues that are relevant to the ES of the food system. Identifying and analyzing these in future studies of this nature would better highlight the complexities of the discourse around ES considerations as well as a more complete framing analysis that includes key stakeholder.

This study piloted a novel method for food and nutrition policy analysis. Our findings have demonstrated its rigor and capacity to uncover the broader socio-political and historical context within which the NFP developed, the number and type of policy actors, degree of support for ES integration and changes to the NFP’s content with respect to ES over time. The methodology would be generalizable to policy-relevant research in other contexts and topic areas. However, it would be important for future research to evaluate the robustness of this method. We also suggest that conducting stakeholder interviews to support a qualitative frame analysis would be a relevant inclusion to the methodology, so as to help reveal the problem definitions and solutions applied by vested interests.

## Conclusion

The initial intentions for ES considerations to be integrated into the NFP were abandoned, resulting in a business-as-usual approach to food and nutrition policy making. Despite widespread and solid stakeholder support for including ES considerations in the NFP, the interests of a relatively small albeit powerful, number of stakeholders coincided with the dominant neoliberal ideology shaping the Australian public policy environment to critically influence the orientation and content of the final policy outcome. Our findings indicate that Australia’s political environment is currently not sufficiently mature for ES considerations to be integrated into food and nutrition policies to a meaningful extent. We observed a lack of transparency in the way that different views were weighted in informing the policy-making process. Given the highly politicized nature and urgency of this issue we propose reforms to the stakeholder consultation process be considered. The proposed consultation reforms would be to provide a modified consensus model promoting transparency and more equitable access to decision making to enable consultation to better manage political influence during the policy-making process. This study demonstrated an effective method for analyzing a food and nutrition policy though future research should consider the robustness of the approach in other policy contexts.

## Author Contributions

ML and JW devised and supervised the study. All authors contributed to the study design. ER collected and analyzed the data. ML and JW validated the results. ER wrote the manuscript with support from ML and JW. All authors read and approved the final manuscript.

## Conflict of Interest Statement

The authors declare that the research was conducted in the absence of any commercial or financial relationships that could be construed as a potential conflict of interest.
